# Ovine and Caprine Brucellosis (*Brucella melitensis*) in Aborted Animals in Jordanian Sheep and Goat Flocks

**DOI:** 10.4061/2010/458695

**Published:** 2010-10-28

**Authors:** Assadullah Samadi, M. MK. Ababneh, N. D. Giadinis, S. Q. Lafi

**Affiliations:** ^1^Faculty of Veterinary Medicine, Jordan University of Science and Technology, P.O. Box 3030, Irbid 22110, Jordan; ^2^Clinic of Farm Animals, School of Veterinary Medicine, Aristotle University, 546 27 Thessaloniki, Greece

## Abstract

Two hundred and fifty five biological samples were collected from 188 animals (81 sheep and 107 goats) during the lambing season from September 2009 to April 2010 from the Mafraq region of Jordan. Sampled animals belonged to 93 sheep and goat flocks that had abortion cases in the region. One hundred and seven (41.9%) biological samples were positive for the *omp2* primers that were able to identify all *Brucella* species in the collected samples which were obtained from 86 aborted animals (86/188 = 45.7%). Using the B. melitensis insertion sequence 711 (*IS711*) primers on the 107 *omp2* positive samples, only 61 confirmed to be positive for *B. melitensis*. These positive samples were obtained from 28 sheep and 33 goats. The prevalence rate of *B. melitensis* was 27.1% (51/188) among aborted animals. For differentiation between vaccine strain and field strain infection, polymerase chain reaction-restriction fragment length polymorphism (PCR-RFLP) method using *Pst*I endonuclease enzyme was used. Vaccination with Rev-1 in the last year (OR = 2.92, CI: 1.1–7.7) and grazing at common pasture (OR = 2.78, CI: 1.05–7.36) were statistically significant (*P* ≤ .05) risk factors positively associated with the occurrence of brucellosis in sheep and goat flocks.

## 1. Introduction

Brucellosis, especially caused by *B. melitensis*, remains one of the most common zoonotic diseases worldwide with more than 500,000 human cases reported annually [1]. *B. melitensis* has 3 biovars (1–3), highly pathogenic for humans [2]. Brucellosis is present throughout the five continents and it is still an uncontrolled serious public health problem in many developing countries [3]. It is endemic in sheep and goats in most countries of the Mediterranean basin, the Middle East, Central Asia [4, 5], with only North America, North Europe, South-East Asia, and Oceania being spared [6]. Animal brucellosis poses a barrier to trade of animals and animal products between countries and causes considerable economic losses due to abortion and fertility problems to the sheep and goat industry [3, 7]. 

Control measures are based on strict hygiene and vaccination programs. Vaccination is regarded as a measure for reducing the prevalence of the disease to a level where eradication by test and slaughter can be implemented. Of the vaccines used for immunizing small ruminants against *B. melitensis*, Rev-1 vaccine is generally preferred [8, 9]. The Rev-1 vaccine is indicated to protect small ruminants against brucellosis and to protect females from abortion in regions where the disease occurs. Conjunctival vaccination is safer than subcutaneous vaccination but is not safe enough to be applied regardless of pregnancy status of animals [10] and the duration of immunity conferred by this method of vaccination is the subject of controversy. 

Serological test, identification of the agent by culture and Polymerase chain reaction (PCR) test are the most common techniques that are used for brucellosis diagnosis [11]. Because of the hazardousness of vaccine strains that can cause disease in human and animals, PCR-RFLP assay is being used successfully to differentiate all vaccine strains from field infection using *omp2* gene of brucellae which has 2 alleles *omp2a and omp2b. *This method can differentiate field infection with Rev-1 vaccines by producing different band pattern using *Pst1* endonuclease enzyme [12]. *Omp2a* does not have the restriction site of *Pst*I and therefore is not a good target for differentiation of vaccine strains with field strain in PCR RFLP but *omp2b* has the mentioned site for the *Pst*I enzyme and can be used successfully for differentiation of all *Brucella* vaccine strains with the field strains infection [12]. 

*B. melitensis *strain Rev-1 has the normal properties of a biovar 1 strain of *B. melitensis*, but develops smaller colonies on agar media; it does not grow in the presence of basic fuchsine, thionin (20 *μ*g/mL), or benzyl penicillin (3 *μ*g/mL) (final concentrations) but does grow in the presence of streptomycin at 2.5 or 5 *μ*g/mL (5 IU/mL) [13]. Vaccine strain Rev-1 may also be identified using specific PCRs [14].

Recently, a robust and rapid multiplex PCR assay has been introduced which allows for the differentiation of all nine currently recognized *Brucella* species including the recently described *Brucella* species, *B. microti, B. inopinata, B. ceti*, and *B. pinnipedialis* [15].

The purposes of this study were: (1) to identify *B. melitensis *in aborted cases (sheep and goats), (2) to estimate the prevalence rate of *B. melitensis *in aborted animals (sheep and goats), (3) to identify and differentiate vaccine strain from field strains infection, and (4) to evaluate some risk factors thought to be associated with the occurrence of *B. melitensis *in sheep and goat flocks in Mafraq region of Jordan.

## 2. Materials and Methods

### 2.1. Study Area, Environment, and Management of the Flocks

Mafraq Governorate, which lies northeast of Jordan, is considered as the most important area for raising animals, specially sheep and goats which are the most abundant domestic animal in this region of Jordan. There were about 3000 flocks that were present in this region in 2009, about 40% of the Jordanian sheep population (Ministry of agriculture records). The main breed of sheep in Jordan is Awassi. Sheep industry is considered an important source of income for people in Mafraq. Sheep flock of this study could be classified as seminomadic because they move out from their original areas for grazing in the Western parts of Jordan during early March until the end of August. During the rest of the year until the following spring season, these flocks were housed and group fed about 1 kg straw, 600 g barely, and 200 g wheat-bran per head per day. 

The flocks of this study were located at 37°.26′–38°.41′E, 31°.38′–32°.27′N at 650–696 m above sea level. The annual rainfall varied between 132 and 220 mm. In January (winter season), the 4 monthly mean maximum and minimum temperature ranged between 5°C to 20°C and −3°C to 7°C, respectively. The monthly mean relative humidity ranged between 20% and 81%. In June (summer season), the monthly mean maximum and minimum temperature ranged between 29°C to 44°C and 12°C to 28°C, respectively. The monthly mean relative humidity ranged between 10% and 40%. 

In the Northern part of Jordan, estrus activity of sheep starts in mid-July of the year and lambing and kidding season in Jordan extends from December to March [16]. Under normal conditions, less than 10% of the sheep lamb twice a year. Usually lambs are weaned at the age of 60–90 days and most ewes are milked twice a day for an average milking period of 120 days and an average milk production of 0.63 liter per day [17].

### 2.2. Study Design, Target Population, and Sample Collection

This study was designed as a convenient cross-sectional study, where the flock was the study unit and the outcome variable was the brucellosis status of the flock, classified as positive or negative. One positive animal in the flock was enough for the flock to be classified as positive. The target population consisted of all goat and sheep flocks that were present in Mafraq area of Jordan. There were 3000 goat and sheep flocks distributed throughout the region [18]. The procedure and formula of Martin et al. [19] were used to calculate the number of flocks needed for this study. All calculations were based on previously published results conducted in the region by Al-Talafhah et al. [20] which revealed a 61% *Brucella* seropositive prevalence between flocks and 14% within flock. Also, we assumed that *Brucella* positive seroprevalence flocks were at higher risk for developing abortions. 

We calculated the sample size (number of flocks) to be sampled from the target population for a finite population with an expected prevalence of 61% and specified precision of 10% of the true prevalence with 95% certainty. The total number of flocks required for this study was 93 flocks. Since the main objective of this study was to study the provenance of brucellosis among aborted animals, only goat and sheep flocks with reported abortion cases were included in this study. We decided to select the 93 flocks from the flocks of which their owners asked for veterinary services or consulted with the Veterinary Services/Ministry of Agriculture in Mafraq governorate to investigate the problem of abortions in their flocks during the lambing season 2009/2010. During a successive lambing season from September 2009 to April 2010, we were able to collect biological samples from only 188 reported animals in these flocks (55 goats and 133 sheep) that had abortions (many sporadic abortion cases in these flocks were not examined because farmers notified the veterinary services when they had epidemics with several case of abortions). 

It was possible to collect only 106 fetuses and 149 blood samples from aborted animals within a week after abortion. Of the 188 aborted animals, 67 animals had paired samples (fetal tissue and blood of their dams obtained from 31 goats and 36 sheep) and 121 aborted animals that had just one sample, either blood (82 animals; 67 goats and 15 sheep) or fetal tissues (39 animals; 9 goats and 30 sheep). 

Whole blood (10 mL in a tube with anticoagulant) was collected from the jugular vein. Different fetal tissues such as kidney, liver, lung, and brain were collected from freshly aborted fetuses during 24 hrs after abortion. All blood and tissue samples were transferred to the Jordan University of Science and Technology (JUST) laboratory using special container with ice and were kept at −20°C until used for DNA extraction and PCR analysis. 

### 2.3. Isolation of DNA from Blood and Fetal Tissue Samples

DNA isolation was performed according to the manufacturer's instruction (DNA Purification Kit, Promega, USA). Briefly, 300 *μ*L of blood sample was used in a 1.5 mL of a sterile eppendorf tube with 900 *μ*L of erythrocyte lysis solution, and incubated at room temperature for 10 minutes, then centrifuged at 16000 rpm using refrigerated eppendurf centrifuge for 1 min. The supernatant was discarded and the leukocyte pellet was resuspended using vortexes for 20 seconds at high speed. On the resuspended white pellet, 300 *μ*L nucleic lysis solution was added and pippetted 3–5 times to lyse the white blood cells (WBCs) and then was incubated at 37°C for one hour and 1.5 *μ*L of RNase solution was added on the mixture and incubated at 37°C for 15–20 minutes. Then 100 *μ*L of protein precipitation solution was added and vortexed for 20 seconds at high speed. Small clump of protein was visible after this step. The lysate was centrifuged at 16000 rpm for 4 minutes at room temperature. A dark brown protein pellet was visible after centrifugation. The supernatant containing total DNA was transferred to a fresh clean 1.5 mL eppendurf tube containing 300 *μ*L room temperature Isopropanol. The solution was gently mixed 5–8 times by inversion until the white thread-like strands of DNA formed a visible mass. DNA was recovered by centrifuging the samples at 16000 rpm for 5 min, and the pellet was rinsed with 300 *μ*L of 70% ethanol, dried and then resuspended in 60 *μ*L of DNA rehydration solution. Extracted DNA was kept at −20°C until used in PCR analysis. The concentration and purity of the DNA was determined spectrophotometrically. Also the quality and quantity of DNA was examined by running 5 *μ*L DNA sample after mixing with loading dye on agarose gel. Sheep Glyceraldehyde 3-phosphate dehydrogenase (sGAPDH) was also amplified by PCR for all extracted DNA samples to confirm the quality of DNA in our extracted DNA samples.

DNA from fetal tissues was extracted as follow: briefly, 1 g pooled sample of different fetal tissues with 3 mL of 1X PBS (phosphate buffer saline) was added in a clean 10 mL white cap tube, and then homogenized using a tissue homogenizer. After homogenization, the lysate was centrifuged at 3000 rpm for 5 minutes at 4°C. Two hundred *μ*L of supernatant was transferred to a clean eppendurf tube containing 600 *μ*L nucleic lysis solution and was pipetted many times to lyse the cells until no clumps of cell was visible. The lysate was incubated at 65°C for 15–30 minutes in the water bath. On the cooled lysate, 3 *μ*L of RNase was added and incubated at 37°C for 15–30 minutes. DNA was then obtained as mentioned before.

### 2.4. Primers

Published *Brucella*-specific primer pairs were used to amplify *Brucella omp*2 gene. These are sequences of the forward 5′TGGAGGTCAGAAATGAAC3′ and reverse 5′GAGTGCG AAACGAGCGC3′ primer pairs. This primer set could identify all species of *Brucella.* A single band with the expected size of 282-bp was obtained with all isolates [12].

The following primer pairs: AAATCGCGTCCTTGCTGGTCTGA and TGCCGATCACTTAAGGGCCTTCAT, specific to *IS711* element of *B. melitensis* were used to confirm *B. melitensis* from aborted fetal tissues and blood samples. The amplified product of this primer set was 731-bp [21].

### 2.5. Polymerase Chain Reaction (PCR)

PCR amplification was performed using Promega Gotaq Green Master Mix (USA) as follow: briefly, the PCR was performed with total volume of 25 *μ*L containing 12.5 *μ*L of Gotaq Green Master Mix, 2 *μ*L of every forward and reverse primers, 2 *μ*L MgCl_2,_ 4.5 *μ*L nuclease free water, and 2 *μ*L of genomic DNA. Following hot start treatment at 94°C for 4 min., PCR was performed as follow: 35 cycles of PCR with 1 cycle consisting of 1 min at 94°C for DNA denaturation, 1 min at 50°C for primers annealing ((for *omp*2 primers set) but 56°C for *B. melitensis* specific primer set) and 1.5 min at 72°C for polymerase-mediated primer extension. The last cycle included incubation of the sample at 72°C for 10 min and was kept at 4°C for unlimited time. Seven microliters of the amplified product was analyzed by electrophoresis in ethidium bromide stained 1.5% agarose gel in TBE buffer. The amplified product was visualized under UV light and then was photographed using Alphalmager (Alpha Innotech) image documentation system. 

### 2.6. Digestion of the Amplified Products

PCR-RFLP is used to differentiate all vaccine strains from field infection using outer membrane proteins2 gene (*omp2*) of brucellae which has 2 alleles; *omp2a *and *omp2b. *This method is able to differentiate field infection with Rev-1 vaccine by producing different band patterns using *Pst*I endonuclease enzyme [12].* Pst*I restriction enzyme was used according to the manufacturer's instruction (Bio labs) [12]. Briefly, the 282-bp band of PCR product of *omp*2 gene was cut from the agarose gel by scalpel. PCR-DNA was purified using Promega Wizard SV Gel and PCR Clean up System (USA) as follow. The band of DNA with the gel was poured in 1.5 mL eppendurf tube and then for every one mg of gel with DNA band, an equal volume of 10 *μ*L membrane binding solution was added and was incubated at 60°C for 10–20 minutes for gel to be dissolved. The dissolved gel then was poured on a specific column membrane and was incubated at room temperature (RT) for 2 minutes then centrifuged at 15000 rpm for one minute. The column membrane was then rinsed twice by washing solution as follow. At first, 700 *μ*L of washing solution was added on the central part of membrane and was incubated at RT for 2 min and then was centrifuged for one min at 15000 rpm. At the second time, 500 *μ*L of washing solution was again added on the central part of membrane and was incubated at RT for one min and then was centrifuged for 5 min as before. For elution of DNA from the column membrane, 15 *μ*L of nuclease free water was added on the central part of membrane and then centrifuged for 1 min as before. 

The 282-bp DNA product of *omp*2 gene that was extracted from the gel was then digested using *Pst*I endonuclease enzyme with total volume of 20 *μ*L as follow: two *μ*L of buffer with 0.2 *μ*L of bovine serum albumin (BSA), 2 *μ*L of *Pst*I enzyme, and 4 *μ*L of purified PCR from the gel, then completed to a final volume of 20 *μ*L with nuclease free water. The mixture was mixed and incubated for 16 hours at 37°C.

### 2.7. Sequencing of 16S Ribosomal RNA (rRNA) and omp2 Product

For better analysis of the origin and antigenic characteristic of *Brucella* species that are present in Jordan, extracted *B. melitensis* DNA and the 282-bp PCR products (*omp*2) were sent for sequencing, 16S rRNA and *omp2 *gene were sequenced in Macrogen Inc., Korea. A phylogenetic tree was built using Lasergene software (Figure 3).

### 2.8. Data Collection

A semistructured questionnaire (written in Arabic language and available upon request from the author) was developed to gather information about management practices. Factors hypothesized to be associated with the risk of brucellosis of sheep and goats were selected after a review of the related scientific literature [11, 22–25]. Questionnaires were administered from September, 2009 to April, 2010. The questionnaire had 23 questions and was grouped into four main management categories: flock health status, reproductive management, nutrition, and other farm-related practices. Seventeen questions were of a closed-ended type with 2 options while 6 questions had 3 options. A pilot testing of the questionnaire was performed on five nonparticipating farmers to identify potential sources for misinterpretation of the questions and to further refine the questions. Flock owners/managers were interviewed in person and through the phone to complete the questionnaires. Each personal interview lasted 20–30 min. All data from the questionnaires were entered into SPSS database, carefully checked and errors were corrected. 

### 2.9. Data Analysis

Data analysis was performed using SPSS 17.0 software for windows (SPSS Inc., Chicago, IL, USA). Associations between the outcome variable (status of brucellosis in the flocks) and its potential risk factors were first screened in a univariable analysis using Chi-square and Fisher exact tests. Potential risk factors with *P *value ≤ .25 (two tailed; *α* = 0.25) which provided that there was no collinearity between variables were then considered for further analysis. Collinearity between the potential risk factors was assessed using *χ* ^2^ test. A multivariable model for the outcome variable was constructed using manual stepwise forward logistic-regression analysis. Risk factors that were not significant in the model were re-entered whenever a new risk factor became significant, or a risk factor was removed. Potential confounders were considered in every model. A risk factor was considered as a confounder if the point estimates of the coefficients in a model changed >10% with the potential confounder present. In the final model, a variable with a *P* value ≤ .05 was considered statistically significant and retained in the model. The fit of the models was evaluated using the Hosmer and Lemeshow goodness-of-fit test [26].

## 3. Results

### 3.1. PCR Results

**Figure 1:**
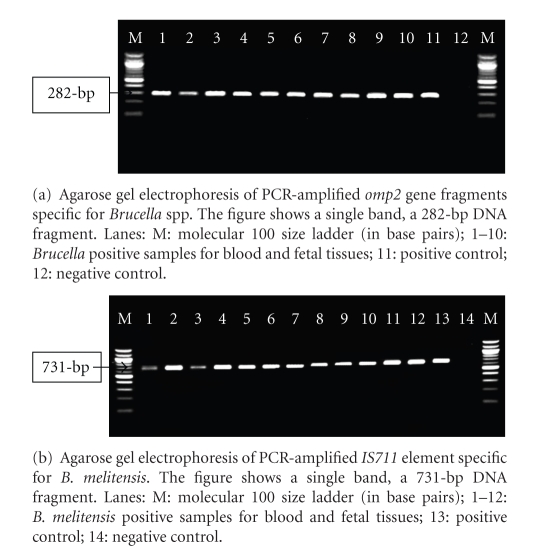


Of the 255 blood and tissue samples collected, 107 (42%) samples were positive for *Brucella*; 51/106 (48.1%) and 56/149 (37.6%) fetal tissue and blood, respectively. These positive samples belonged to 86 aborted animals (86/188 = 45.7%). To confirm the presence of *B. melitensis* in the 107 positive samples, PCR was utilized using *B. melitensis* specific primer for its *IS711* element, 61 biological samples were positive (34 tissue and 27 blood). These samples belonged to 26 goats and 25 sheep (Figures 1(a) and 1(b)). The purified PCR product sequencing result along with 16S rRNA gene of *B. melitensis *confirmed our isolates to be *B. melitensis* (Figure 3). The nucleotide similarity between the 16 *Brucella spp*. is 100% including our *B. melitensis* isolate.

### 3.2. PCR-RFLP (PstI Enzyme)

**Figure 2:**
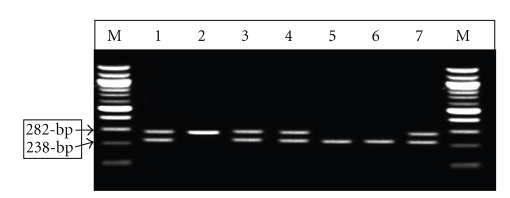
Agarose gel electrophoresis of *Pst*I digests of amplified *omp2 *gene fragments from *Brucella *spp. The figure shows the uncut 282-bp DNA and the larger, *Pst*I-digested (238-bp) DNA fragments. The smaller 44-bp DNA fragment is not shown. Lanes: M: molecular 100 size ladder (in base pairs); 1, 3, and 4: *B. melitensis* Rev-1 like isolates (282 and 238-bp); 2: uncut *B. melitensis *isolates (282-bp); 5 and 6: *B. melitensis* field strain isolates (238-bp); 7: *B. melitensis *Rev-1 vaccine as a positive control.

**Figure 3:**
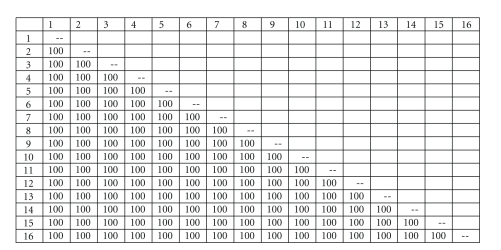
Nucleotide similarity of 929 nucleotides of 16s rRNA gene of *Brucella *spp. 1: *B. abortus* S19, 2: *B. canis*, 3: *B. cetaceae*, 4: *B. melitensis biovar abortus*, 5: *B. melitensis biovar neotomae*, 6: *B. melitensis biovar abortus*, 7: *B. melitensis biovar canis*, 8: *B. melitensis biovar suis*, 9: *B. melitensis biovar-1*, 10: *B. melitensis*-Jordan, 11: *B. melitensis*, 12: *B. melitensis biovar ovis*, 13: *B. microti*, 14: *B. ovis*, 15: *B. pinnipedialis*, 16: *B. suis*.

The sixty one positive samples for *B. melitensis* specific primers were used for RFLP analysis to differentiate Rev-1 vaccine strain from the field strains infection. Thirty three (54%) of samples had vaccine strain patterns while 2 samples (3.27%) had field strain pattern, but 26 samples (42, 6%) were not cut by the *Pst*I enzyme in RFLP analysis (Figure 2). An example of the Rev-1 RFLP pattern is lanes 1, 3, and 4, the field pattern represented in lane 5 and 6, and the uncut *B. melitensis* pattern as in lane 2. Rev-1 vaccine DNA was extracted and was used as a positive control in PCR-RFLP analysis. 

### 3.3. Prevalence Rate of *B. melitensis* in Aborted Animals

The crude prevalence rate of brucellosis was 27.1% (number of brucellosis positive aborted cases during the study period/total number of aborted animals during study period × 100) among aborted animals in Mafraq region of Jordan. There were 32 (34.4%) flocks that had at least one *B. melitensis* positive samples.

### 3.4. Statistical Analysis

Of the 93 sampled flocks that were used in this study, we were able to fill the questionnaires for 89 farms (95.7%) during visits or through phone calls. The other 4 flocks changed their location and we were not able to contact them for filling the questionnaires. Therefore, data of the remaining 89 flocks was used in the analysis of this study. A total of 20 variables were screened in the initial univariable analysis, 3 had their *P* value ≤ .25 and were considered for further analysis. These three variables were offered to construct the final logistic regression model. Two variables remained in the final multivariable model with a *P* value ≤ .05 (Table 1). Vaccination with Rev-1 and grazing at common pasture were the only variables that had *P* value *<* .05. Presence of dog was identified as a confounder variable and was forced in the final model. 

## 4. Discussion

This is the first molecular study of ovine and caprine brucellosis in Jordan that estimated the prevalence rate of brucellosis in aborted animals and evaluated the risk factors that were hypothesized to be associated with the occurrence of this disease.

Our study revealed that the prevalence rate of brucellosis among sheep and goat flocks was in close agreement with a previous report conducted in the region by Al-Talafhah et al. [20] indicating that the brucellosis is an endemic health problem of sheep and goats over the past decade in the region. Since it has been done by many other researchers in the same place and its rate in serology have been reported to be between 45–56 % in sheep and goat flocks using RBT and ELISA tests [27]. 

Genus specific primers (*omp2*) showed that 41.9% (107/255) of biological samples (blood and tissue) were positive but 23.9% (61/255) of them were just positive for *B. melitensis* specific primers (IS711). Al-Majali [27] reported the presence of *B. abortus* biotype 9 in infected goats with brucellosis in the same region. This might be the reason of different positive results in two different primers (Genus specific primers and IS711, specific for *B. melitensis*).

PCR-RFLP and multiple logistic regression results revealed that the majority of positive cases of *B. melitensis* had Rev-1 vaccine strain pattern and vaccination with Rev-1 last year was one of the main risk factors associated with the positivity of the flocks with brucellosis. This might be due to the improper use of Rev-1 vaccine in Jordan as many farmers continue to vaccinate their animals annually regardless of their pregnancy status. Sheep and goats are vaccinated annually by SC rout in Jordan with full dose vaccine of Rev-1 live attenuated strain with no age limitations. Although the vaccine has not to be used during pregnancy, but some of the farmers may use it even during pregnancy because many time the vaccine is administrated by the farmer itself and many of them are illiterate. It has been reported that this vaccine is not safe if it is used during pregnancy even with reducing doses subcutaneously or conjunctively [10, 11, 28–30]. Recently, Kojouri and Gholami [25] reported that bacteremia can be prolonged for more than 60 days after vaccination with Rev-1 vaccine and has the ability of dissemination from vaccinated animals to the healthy ones. However, further field experimental study is essential to verify the efficacy and the hazard associated with Rev-1 vaccine in Caprine and Ovine population of Jordan.

 Our result of RFLP showed that 26 positive *B. melitensis* samples were not cut by P*stI* enzyme. This might be due to the ability of *B. melitensis* to go through genetic diversions in the *omp2* gene (a & b) [12], and this might be the reason behind the uncut pattern. 

Grazing at common pasture was also a potential risk factor for brucellosis in sheep and goats farms. Similar results were reported previously in the same region [27] and elsewhere in the world [22, 23, 31], as it has been reported that mixing herds at pasture and keeping the animals in shelters during the night, represent major risk factors for transmission of the infection [23]. 

Presence of dogs in the farms or with the flocks was considered a potential confounder factor that may increases the chance of *Brucella* infection for the animal. In this study, the majority of the farmers (60.7%) gave the aborted fetuses to their dogs that were present with their flocks permanently, and possible reason is that, the dogs can be infected with *B. melitensis* and Rev-1 vaccine strains and subsequently transmit the infection to the farm animals through excretions or mechanically [1, 23, 32]. 

## 5. Conclusions

Previous reports and our result confirm that brucellosis is an endemic disease in small ruminant flocks in Mafraq/Jordan. The number of cases of *B. melitensis* is relatively high among aborted animals. It is considered as an important cause of abortion in these species. Improper use of Rev-1 vaccine and grazing at common pasture and presence of dogs in the flock were significant risk factors associated with the occurrence of this disease in sheep and goat flocks.

## Figures and Tables

**Table 1:** Final logistic regression model for risk factors associated with the *B. melitensis* positivity of small ruminant flocks in Mafraq region of Jordan.

Variable	b	S.E._b_	*P*-value	OR	95% CI for OR
Vaccination by Rev-1 in the last year					
Yes	1.07	0.49	0.03	2.9	1.1, 7.8
No	Ref.	—	—	—	—
Grazing at common pasture					
Yes	1.02	0.49	0.04	2.8	1.1, 7.4
No	Ref.	—	—	—	—
Presence of dog in the farm					
Yes	0.31	0.48	0.51	1.4	0.53, 3.5
No	Ref.	—	—	—	—
Constant	1.91	0.59	0.00		
